# Accelerating functional MRI using fixed‐rank approximations and radial‐cartesian sampling

**DOI:** 10.1002/mrm.26079

**Published:** 2016-01-17

**Authors:** Mark Chiew, Nadine N. Graedel, Jennifer A. McNab, Stephen M. Smith, Karla L. Miller

**Affiliations:** ^1^ FMRIB CentreUniversity of OxfordOxfordUnited Kingdom; ^2^ R.M. Lucas Center for ImagingStanford UniversityStanfordCaliforniaUSA

**Keywords:** fMRI, k‐t acceleration, low‐rank acceleration, matrix completion, k‐t FASTER, golden ratio

## Abstract

**Purpose:**

Recently, k‐t FASTER (fMRI Accelerated in Space‐time by means of Truncation of Effective Rank) was introduced for rank‐constrained acceleration of fMRI data acquisition. Here we demonstrate improvements achieved through a hybrid three‐dimensional radial‐Cartesian sampling approach that allows posthoc selection of acceleration factors, as well as incorporation of coil sensitivity encoding in the reconstruction.

**Methods:**

The multicoil rank‐constrained reconstruction used hard thresholding and shrinkage on matrix singular values of the space‐time data matrix, using sensitivity encoding and the nonuniform Fast Fourier Transform to enforce data consistency in the multicoil non‐Cartesian k‐t domain. Variable acceleration factors were made possible using a radial increment based on the golden ratio. Both retrospective and prospectively under‐sampled data were used to assess the fidelity of the enhancements to the k‐t FASTER technique in resting and task‐fMRI data.

**Results:**

The improved k‐t FASTER is capable of tailoring acceleration factors for recovery of different signal components, achieving up to R = 12.5 acceleration in visual‐motor task data. The enhancements reduce data matrix reconstruction errors even at much higher acceleration factors when compared directly with the original k‐t FASTER approach.

**Conclusion:**

We have shown that k‐t FASTER can be used to significantly accelerate fMRI data acquisition with little penalty to data quality. Magn Reson Med 76:1825–1836, 2016. © 2016 The Authors Magnetic Resonance in Medicine published by Wiley Periodicals, Inc. on behalf of International Society for Magnetic Resonance in Medicine. This is an open access article under the terms of the Creative Commons Attribution License, which permits use, distribution and reproduction in any medium, provided the original work is properly cited.

## INTRODUCTION

Functional MRI (fMRI) is increasingly taking advantage of acceleration methods to improve the efficiency of data acquisition, increase achievable temporal and spatial resolutions, and reduce artifacts (e.g., image distortion or physiological noise). Higher sampling rates in fMRI increase the temporal dimensionality of the time‐series data, which is beneficial for statistical inference 1. Moreover, they provide an opportunity for finer characterization of hemodynamic response features. Alternatively, interest in using fMRI to probe functional organization of the brain at finer scales, like cortical layers 2 or columns 3, particularly combined with ultrahigh field MRI at 7 Tesla (T) or above, incurs a significant burden on acceleration to mitigate longer volume repetition times (TRs) at high matrix sizes.

Conventionally in fMRI, acceleration is achieved using parallel imaging techniques such as SENSE 4 or GRAPPA 5, which rely only on spatial coil sensitivity information resolve undersampling. More recently, simultaneous multislice or “multiband” methods 6, 7, which accelerate by acquiring more than one slice at a time without sampling reduction, and three‐dimensional (3D) echo planar imaging (EPI) methods have seen increased adoption 8, particularly when combined with CAIPIRINHA 9 to mitigate signal‐to‐noise ratio (SNR) loss. However, these methods only use spatial information in images from single time‐points, independently. While these methods are powerful, they do not exploit information contained in the time domain. Parallel imaging approaches that do incorporate information across temporal frames, such as k‐t GRAPPA 10, do so by enforcing strong temporal autocorrelation, which can undermine the goal of increasing temporal resolution.

Recently, we introduced a method, k‐t FASTER (fMRI Accelerated in Space‐time by means of Truncation of Effective Rank) 11, that explicitly leverages the fact that fMRI data have a limited number of spatial and temporal components that contain the signals of interest. In other words, fMRI k‐t matrices can be robustly characterized with fixed rank approximations, which permits the application of techniques developed for recovery of undersampled matrices 12. These matrix completion methods are similar to compressed sensing 13, where matrix rank constraints replace vector sparsity constraints, although with our approach no prespecified sparsifying basis is needed, as fMRI data are not sparse under conventional transforms. Our original work demonstrated the ability for k‐t FASTER to robustly accelerate resting fMRI data acquisition by just over 4 × (acquiring only 23% of k‐space) by using an undersampled multishot 3D EPI sampling strategy combined with an iterative hard threshold and matrix shrinkage reconstruction. Notably, the matrix recovery was performed in a coil‐independent manner to demonstrate the entire burden of reconstruction being carried by the rank constraints.

In previous methods that use temporal information for acceleration in fMRI, the data were required to possess specific spatial‐spectral structure to avoid x‐f aliasing 14, or to permit temporal sparsity‐constrained reconstructions 15. These methods are typically restricted to data with x‐f structure, which limits their applicability. Recent work has also explored the use of Karhunen‐Loeve transform (KLT) to identify a more general sparsifying basis 16, 17; however, these methods require additional training data or use only a subset of acquired data for basis estimation.

In contrast, low rank matrix completion only requires the assumption that the data contain a small number of high‐variance rank‐1 components (relative to the matrix size). In the case of fMRI, a component is described by the outer product of a spatial map and its associated time‐course, representing brain networks (or nuisance signals similar to those identified by independent component analysis). No a priori characterization of those components is necessary. This powerful property circumvents the requirement for knowledge of a sparsifying transform or the need to impose model structure on the matrix estimates.

Preliminary reports of similar methods for rank‐based acceleration of fMRI data acquisition have included additional sparsity constraints to regularize the reconstruction, such as promoting sparsity of the recovered spatial components 18 or sparsity of the images in the wavelet domain 19. In contrast, the k‐t FASTER approach relies solely on a strict fixed‐rank constraint, without relying on prior information or any specific spatial or temporal structure.

Here we introduce an improved version of our k‐t FASTER method, which uses a 3D hybrid radial‐Cartesian sampling trajectory that enables acceleration factors to be selected posthoc. Additionally, we incorporate coil sensitivity maps into the reconstruction in an approach that estimates a single, underlying x‐t matrix. We show, in both numerical simulations and experiments, that the more sophisticated sampling and sensitivity encoding both help to increase achievable acceleration factors, and that acceleration limits are dictated by the strength or variance of components to be recovered in the fixed‐rank signal model, relative to noise. Furthermore, we demonstrate that k‐t FASTER can robustly identify both task activation and intrinsic networks in task experiments up to R = 12.5 and R = 5, respectively, with no prerequisite knowledge of task waveforms, specific design constraints or assumptions of spatial structure necessary for reconstruction.

## METHODS

### Sampling

**Figure 1:**
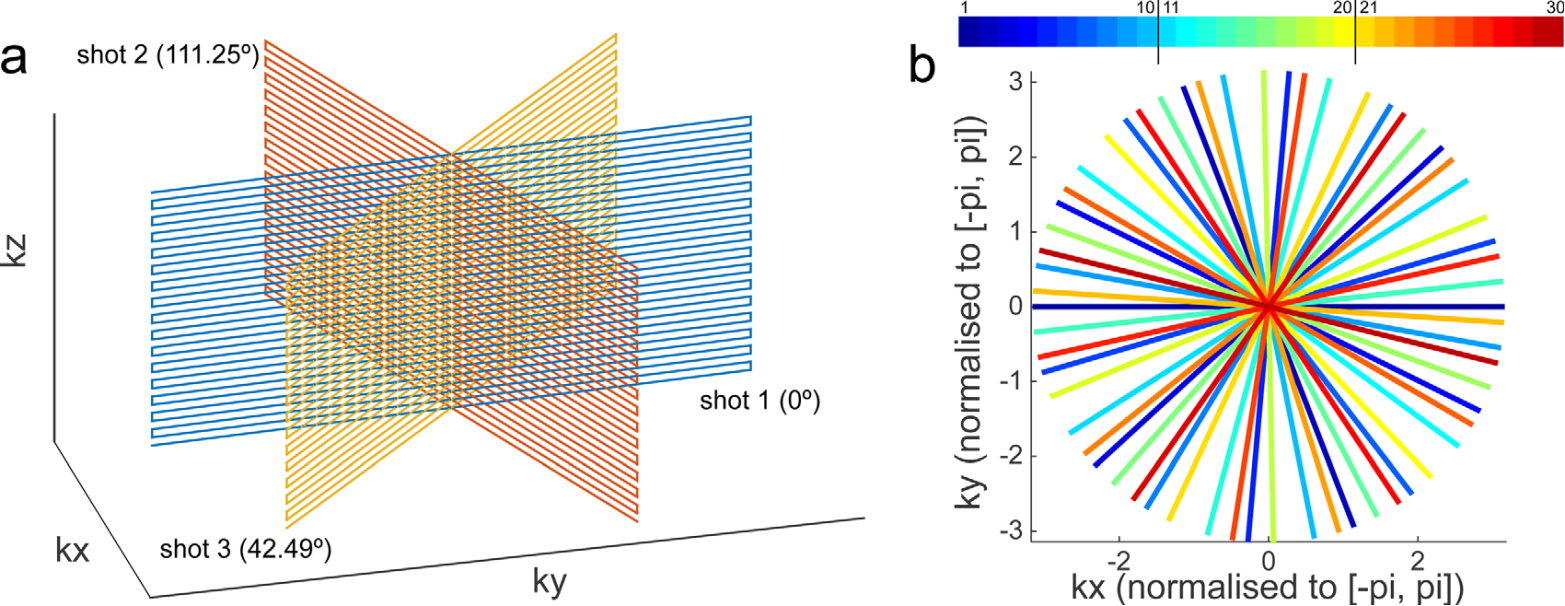
Diagram of the 3D hybrid radial‐Cartesian multishot sampling scheme (**a**). Each shot constitutes an EPI readout with kz phase encoding, and is rotated by approximately 111.25 º about the z‐axis from the shot previous. Top down view along the kz‐axis showing the radial order of acquisition for the first 30 projections (**b**), where projections acquired close together in time share similar color.

The sampling strategy used here comprises a multishot 3D acquisition along a hybrid radial‐Cartesian k‐space trajectory 20, 21, 22 (Fig. 1a). In this approach, each shot samples a 2D plane of k‐space using an echo‐planar trajectory. However, instead of sampling parallel planes in 3D space (as is done in 3D EPI), each plane is rotated about the EPI phase‐encoding direction to cover a cylinder in k‐space, with a Cartesian sampling distribution along k_z_, and radial distribution in k_x_‐k_y_. This trajectory was previously proposed in the context of diffusion imaging and acquired projections with monotonically increasing angle 20, our new implementation sets the angular increment by the golden ratio (180 º/1.618 ≈ 111.25 º). This sampling scheme results in an ordering of radial projections such that any arbitrary number of consecutive measurements produce a near optimal k‐space encoding efficiency 23 (Fig. 1b). This sampling pattern is similar to the “stack‐of‐stars” trajectories used in other dynamic rank‐constrained accelerated acquisition methods 24. However, acquiring data along the z‐direction using an EPI readout instead of line‐by‐line is more efficient for fMRI given the long echo time constraints for optimal BOLD contrast.

One benefit of this sampling scheme is that acceleration factors can be chosen for the data posthoc, so data can be reconstructed at multiple temporal resolutions. While Cartesian sampling patterns fix the acceleration factor of the reconstructed data at acquisition time, this scheme provides more flexibility and potentially increases the utility of the acquired data, permitting second level optimization on temporal bin width. With the richness of fMRI data, signal models of varying ranks can represent different amounts of desired network information, and can be estimated with maximum acceleration factors linked to model order. Previous work with bit‐reversed multiecho radial ordering schemes 25 offer similar benefit, but with reduced flexibility due to the fact that optimal k‐space coverage is achieved only when bin sizes are powers of two.

Another advantage of this sampling procedure, hereafter referred to as “radial” for simplicity, is that the undersampling is distributed across two spatial dimensions, compared with just one dimension in our previous 3D EPI implementation with undersampling in k_z_ only. The artifacts resulting from radial under‐sampling are also less coherent in the image domain compared with more concentrated artifact power resulting from Cartesian under‐sampling schemes. However, radial encodings are inherently less efficient than Cartesian encodings, by a factor of Π/2 (∼57%) 26. To accurately reflect the impact on achievable volume scan times compared with conventional acquisitions, we report all acceleration factors relative to the amount of sampling in an equivalent fully sampled Cartesian acquisition. By this convention, a radial trajectory would have to use some under‐sampling to achieve an acceleration of R = 1.

### Reconstruction Algorithm

Rank constrained optimization problems can be formulated in several different ways. The iterative hard thresholding (IHT) or projected Landweber algorithm 27 solves the following constrained optimization problem using a fixed rank constraint:

(1) $$\begin{array}{c} \underset{X}{\min}\left\|y-\Phi {\left(X\right)}_{2}^{2}\right\|\;such\;that\;rank\left(X\right)=r. \end{array}$$

However, our recent work 11 found that incorporating a matrix shrinkage step improved reconstruction fidelity in fMRI data. Our method of iterative hard thresholding with matrix shrinkage (IHT+MS) is also similar to a variant of the fixed point continuation (FPC) algorithm 28, the latter of which solves the unconstrained problem:

(2) $$\underset{X}{\min}\frac{1}{2}{\left\|y-\Phi (X)\right\|}_{2}^{2}+\lambda {\left\|X\right\|}_{*}.$$

In both Eqs. (1) and (2), X is the estimate of the data matrix, Φ is the measurement and sampling operator, and y is a vector containing the measured data. In Eq. (2), λ is a Lagrange multiplier, and ${\left\|\cdot \right\|}_{*}$ is the nuclear norm (sum of singular values), which is the convex relaxation of the rank constraint.

As the IHT+MS approach is a heuristic variant of the IHT and FPC algorithms, it does not solve Eq. (1) or (2) directly, although descriptions of convergence properties and recovery bounds for the FPC variant exist 29. However, in essence, IHT+MS can be viewed as finding near optimal rank‐ r matrix approximations. This fixed‐rank approach has proven more successful in fMRI data than methods using singular value thresholding heuristics to solve Eq. (2), due to the shallow decay behavior of singular values in fMRI data 11.

**Figure 2:**
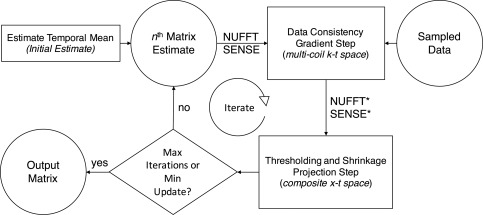
Overview of the IHT+MS matrix reconstruction process. Data consistency is enforced in the multicoil, non‐Cartesian k‐t space, whereas the rank thresholding and shrinkage, and the data estimates are in a composite x‐t space. Adjoint operations are denoted with “*”.

The IHT+MS reconstruction program (Fig. 2) can be summarized as:

(3) $$\begin{array}{l} Y^{n+1}=X^{n}+\mu \Phi^{*}\left(y-\Phi X^{n}\right)\\ X^{n+1}=S_{r,c}(Y^{n+1}) \end{array}$$

where $X^{n}$ is the $n^{th}$ matrix estimate, μ is a step size parameter, Φ is the measurement operator (discussed below) and $S_{r,c}$ is the hard thresholding and shrinkage operator (or projection operator). $S_{r,c}$ has input parameters r, the hard rank constraint, and 0≤c≤1, the matrix shrinkage factor.

Compared with the original k‐t FASTER formulation, the measurement operator Φ=FE is now the composition of the nonuniform Fast Fourier Transform (NUFFT)^30^ and complex coil‐sensitivity encoding, represented by F and E, respectively. With time‐varying sampling, Φ is a general linear operator, not a matrix, and because Φ includes a Fourier transform, the data are estimated directly in the x‐t domain. This formulation facilitates the enforcement of data consistency across arbitrarily sampled k‐space and across multiple coils, which are the two improvements made over the single‐channel, Cartesian sampling approach originally demonstrated. It can be viewed as either a rank‐regularized iterative SENSE reconstruction 31, or as the analogue to distributed compressed sensing 32. In the former interpretation, the rank constraint effectively suppresses noise‐like, residual incoherent aliasing, whereas in the latter, the multiple coils increase the number of effective measurements.

### Coil Sensitivity Estimation and Coil Compression

In both simulations and experiments, coil sensitivity profiles were estimated using the adaptive combine method 33, taking the complex coil combination weights at each voxel as the sensitivity values. Rectangular neighborhoods extending four voxels in each direction were used for weight estimation in a given voxel, and coil sensitivities were arbitrarily phased relative to the phase of coil channel 1. To generate the images required by the sensitivity estimation procedure, the data from all radial projections were averaged, effectively performing a temporal mean across the undersampled data. Due to the radial sampling, the full set of projections constitute a more‐than‐fully sampled k‐space, and the inverse NUFFT was directly applied to generate mean images. To avoid phase cancellation from the complex temporal averaging, global phase variation was removed before data reconstruction, using a simple regression on the center of k‐space that is sampled every shot.

An SVD‐based coil compression transform 34 was used to reduce the coil dimensionality from 32 to 8 in the experimental data. This reduces the memory burden of the reconstruction while retaining over 90% of the sensitivity variance in all cases. In the simulations, sensitivities from one of the experimental datasets were used.

### Simulations and Experiments

Simulations using retrospectively resampled resting state fMRI data were performed to evaluate the benefits of non‐Cartesian sampling and multicoil reconstruction with knowledge of ground truth. The whole‐brain data used for retrospective resampling were acquired in a single subject using simultaneous multislice EPI at 3T, with a volume TR = 836 ms using a MB = 8 acceleration, and 2 mm isotropic spatial resolution 35. The full image dimensions were 100 × 100 × 64 with 1000 time points. Multicoil k‐space data were generated by multiplying the source image time‐series by coil sensitivities, and using the NUFFT to sample the data in k‐space according to the hybrid radial scheme depicted in Figure 1.

To demonstrate the difference in acceleration efficiency, the radial sampling at acceleration factors of R = 6.67, 8.33, and 12.50 were compared with 3D EPI sampling at R = 4.27, which was the acceleration limit observed in our previous work due to the chosen rank constraint, sampling scheme and absence of coil encoding. Additionally, one to eight virtual coils were used to evaluate varying amounts of coil sensitivity information, with the 3D EPI reconstructions using the same sensitivity encoding scheme described above. This first set of simulations contained perfectly correlated noise across coils to examine the effects of coil sensitivity differences without the added benefit of noise averaging. In the second set of simulations, the radial sampling was similarly evaluated, but with independent noise on each of the 32 input coils channels. All reconstructions used a rank thresholding constraint of 128.

fMRI experiments were conducted using two different tasks to demonstrate the robustness of k‐t FASTER in recovering fMRI signal components in vivo. All data were acquired with informed consent in accordance with local ethics, at 3T using a 32‐channel head coil (Siemens Healthcare, Erlangen, Germany), with a 2‐mm isotropic whole‐brain acquisition with TR/TE = 50/30 ms. Additionally, to ensure that acquisition TE values were short enough for optimal BOLD contrast while minimizing image distortion, parallel imaging acceleration was applied along the EPI direction in all acquired radial data, and was recovered independently of and before the rank‐constrained reconstruction. For the radial data, k‐t FASTER reconstruction was performed independently on 2D slices after inverse Fourier transform along the EPI direction. This allowed the slices to be processed in parallel, reducing overall computation time and memory requirements.

The first experiment was performed in three subjects, with a 5‐min acquisition using a set of 200 projection angles repeated 30 times. The subjects performed a 30‐s off/on block‐design visual‐motor task involving simultaneous bilateral finger tapping and viewing of a 10 Hz flashing checkerboard stimulus. To assess the reconstruction fidelity across a broad range of accelerations, data were reconstructed at acceleration factors of R = 2.5, 3.33, 5, 6.67, 8.33, 10, 12.5, and 16.67 to achieve volume TRs of 2, 1.5, 1, 0.75, 0.6, 0.5, 0.4, and 0.3 s, respectively. In one subject, acceleration factors of R = 20, 25, and 33.33 corresponding to volume TRs of 250, 200, and 150 ms were also tested.

A second experiment using a category fluency task was acquired in an additional 2 subjects, using the same acquisition parameters as experiment 1. This task also used a 30‐s off/on block design, where categories were presented visually (e.g., “tools”), and subjects were asked to think of as many words belonging to the category as possible in the block duration (e.g., “hammer”).

For the task data (spatial dimension 100 × 100 × 72, temporal dimension $6000/(\frac{100}{R})$), rank‐constraints of 32 were used, as task components were observed to be well characterized at this dimensionality. To assess the impact of the choice of rank, one subject was reconstructed at ranks of 8, 16, 32, 64, and 128. The algorithm step size μ was set to 0.1, the matrix shrinkage parameter c was set to 0.5, and 100 iterations were chosen for the simulations and 50 iterations for in vivo data reconstructions, unless the minimum update threshold of 10^‐4^ stopped iterations earlier. In all cases, all time‐points were initialized to the mean image calculated from all projections as described above. For the R = 25 and 33.33 data, μ was set to 0.05 with 100 iterations to ensure convergence. While density compensation weighting is not necessary, simple radial weighting by |k| was used to speed up convergence.

### Data Analysis

For the simulation data, matrix error compared with ground truth was evaluated using the relative Frobenius norm (normalized root‐mean‐square error):

$$err=100\frac{{\left\|\hat{X}-X\right\|}_{F}}{{\left\|X\right\|}_{F}}\%$$

For the in vivo data, FEAT 36 was used to perform a standard GLM (general linear model) regression analysis of the task fMRI data with no additional spatial smoothing. Additionally, model‐free exploration of the reconstructed data was performed using independent component analysis (ICA) with MELODIC 37. All z‐statistic maps were corrected using mixture modelling to ensure correct null distributions (zero mean, unit variance) 37, which is important when comparing across different acceleration factors given that autocorrelation can inflate statistical estimates 38.

## RESULTS

The results from the simulations show that matrix reconstruction error decreases with the new radial sampling strategy compared with under‐sampled 3D EPI, even at much higher acceleration factors (see Table 1). For comparison with the original k‐t FASTER method, a 3D EPI coil‐by‐coil reconstruction with sum‐of‐squares combination at R = 4.27 in the correlated noise data, produced the highest error of 0.059. At acceleration factors nearly three‐fold higher, radial sampling produced matrix estimates with significantly lower error when taking advantage of additional coil encoding. Similarly, the results in Table 1 show that error decreases monotonically with increasing coil information. Supporting Figure S1, which is available online, shows results of a conjugate gradient SENSE reconstruction at R = 6.67 with reconstruction error of 0.113, highlighting the benefit of combined sensitivity encoding and rank constraints.

**Table 1:** Retrospectively Undersampled Simulation Relative Frobenius Norm Errors with Correlated Coil Noise (Top) and Independent Noise (Bottom) across a Range of Sampling and Virtual Coils

Correlated Noise	3D EPI R=4.27	Radial R=6.67	Radial R=8.33	Radial R=12.50
1 Virtual‐coils	5.58%	6.04%	6.72%	8.03%
2 Virtual‐coils	5.52%	4.01%	4.46%	5.43%
4 Virtual‐coils	5.07%	3.43%	3.83%	4.71%
8 Virtual‐coils	4.90%	3.07%	3.41%	4.21%
				
Independent Noise		Radial R=6.67	Radial R=8.33	Radial R=12.50
1 Virtual‐coils		6.56%	7.07%	8.44%
2 Virtual‐coils		3.95%	4.43%	5.43%
4 Virtual‐coils		3.33%	3.75%	4.68%
8 Virtual‐coils		2.91%	3.28%	4.14%

With a single virtual coil, the lowest error was found for the 3D EPI at the lowest acceleration factor of R = 4.27. However, as more virtual coils are added, the relative benefit of the additional sensitivity information is marginal in the undersampled 3D EPI, whereas errors in the radial data decrease by nearly half when the number of virtual coils increased from one to eight. Without multiple coils, the acceleration factor appears to be the dominant factor dictating reconstruction error. However, the impact of having radial undersampling across two dimensions (k_x_ and k_y_), compared with only k_z_‐undersampling in the 3D EPI becomes apparent when looking at multiple coil reconstructions. With independent noise across coil channels, a similar effect of acceleration factor and coil information can be seen, with added benefit at higher numbers of virtual coils due to additional noise averaging.

**Figure 3:**
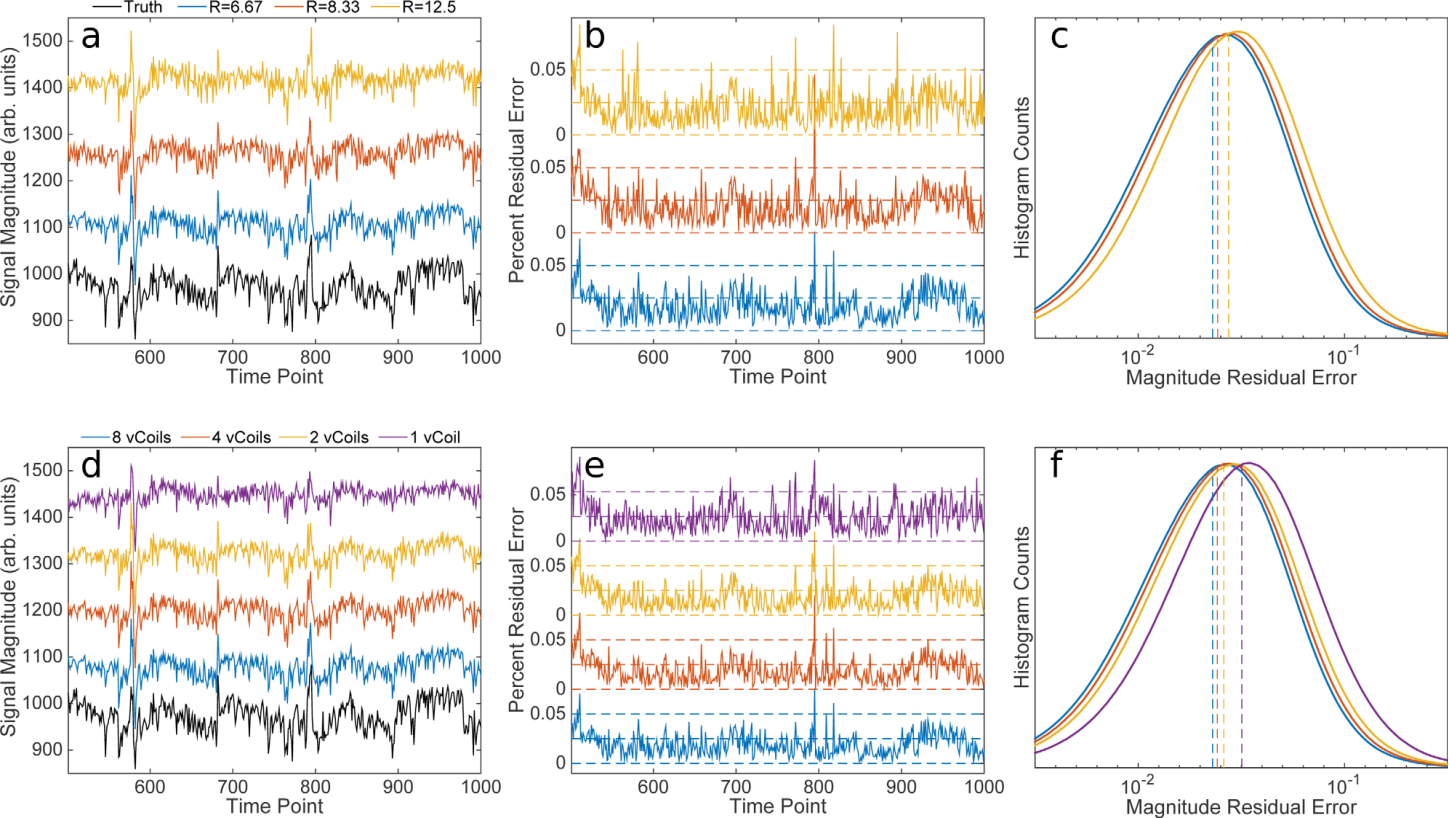
Time‐series segment from a representative voxel across three different acceleration factors R = 6.67, 8.33, and 12.5 (with eight virtual coils), in the radially sampled data (**a**), with corresponding residual errors in (**b**), and log‐transformed error histograms in (**c**). The same voxel time segment, with one, two, four, and eight virtual coils (at R = 6.67) (**d**), and errors in (**e,f**). Dashed lines in (b,d) denote 0%, 2.5%, and 5% error levels, and median error levels in (c,f).

Figure 3 show time‐series from representative voxels in the retrospective sampling reconstructions with independent noise, highlighting the fidelity with which the k‐t FASTER reconstruction is able to recover temporal information. Figure 3a shows the radial datasets at all three acceleration factors (and eight virtual coils). Reconstruction fidelity decreases with increasing acceleration, and is particularly evident in the residual error plotted in Figure 3b, and in the brain‐masked residual error histogram in Figure 3c. Similarly, Figures 3d–f highlight the significant benefit of higher numbers of virtual coils, as expected.

**Figure 4:**
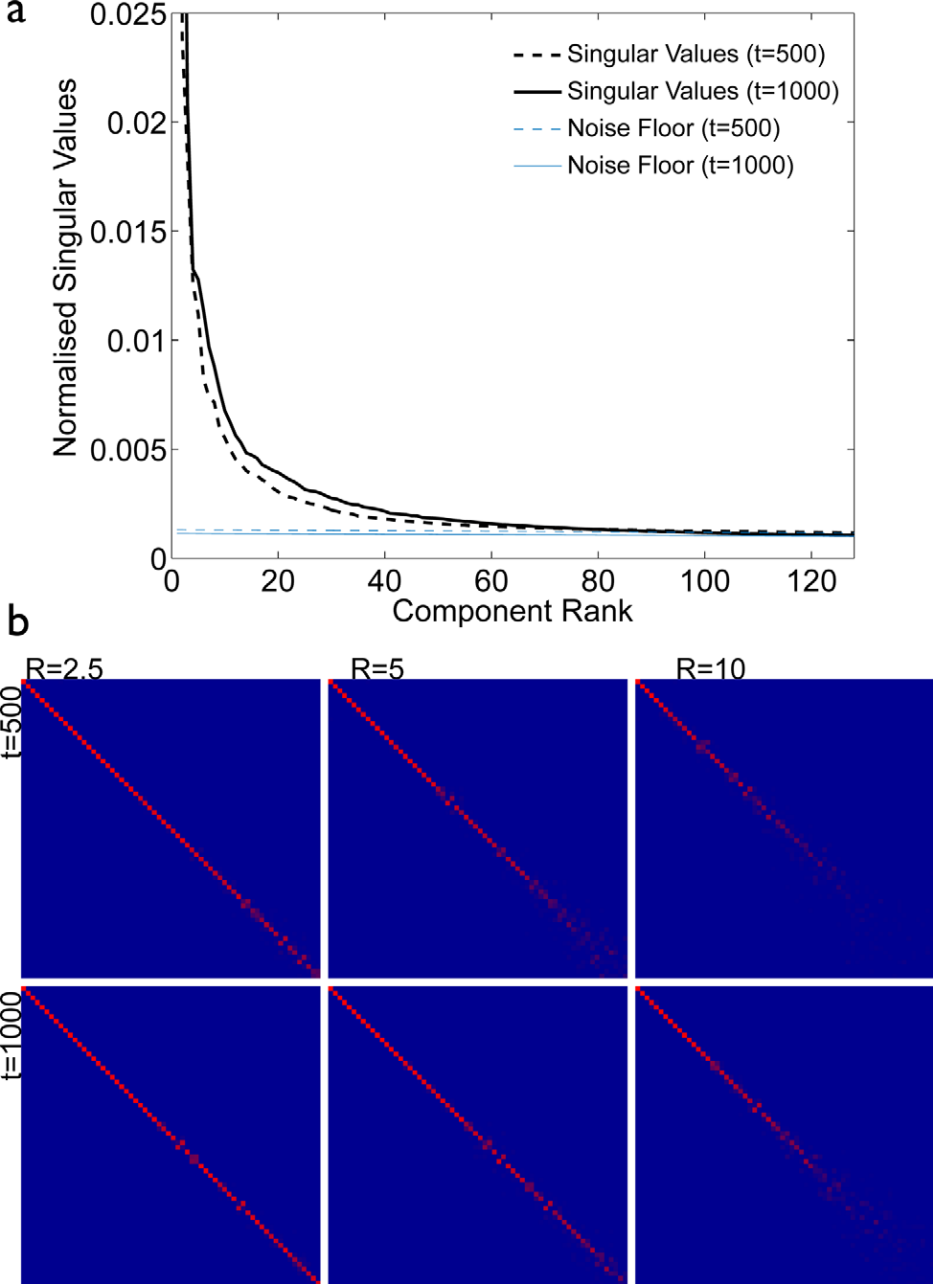
**a**: Distribution of singular values from a ground truth dataset with 500 or 1000 time‐points. **b**: Correlation matrices comparing the first 64 ground‐truth PCA spatial components with the first 64 k‐t FASTER estimated spatial components for the 500 point data (top row) and 1000 time‐point data (bottom).

The fidelity of the estimated spatial subspace was assessed by comparing it to the principal component analysis (PCA) subspace of ground truth data with a typical singular value distribution (Fig. 4a). Two things are apparent from the correlation matrices (Fig. 4b): (i) the number of well recovered basis components decreases with acceleration, and (ii) at the same relative undersampling factor, more components are recovered with increasing time points (imaging duration). Furthermore, the highest relative variance components are well captured at even the highest acceleration factors, indicating that reconstruction errors reflect the systematic loss of lower variance components, rather than uniform loss of fidelity across all components. This is consistent with the link between undersampling factors and the maximum dimensionality of the matrix that is recoverable, where rank‐ r signal models requires at minimum r(m+n−r) samples for unambiguous characterization. Recovery of higher dimensional signal models necessitates greater sampling (reduced acceleration), although the maximum tolerable under‐sampling at any given rank will be case‐specific and depends on the precise distribution of component variances. However, one‐to‐one component mapping is not necessary for subspace identification, and these results do not capture the entire range of signal recovery.

**Figure 5:**
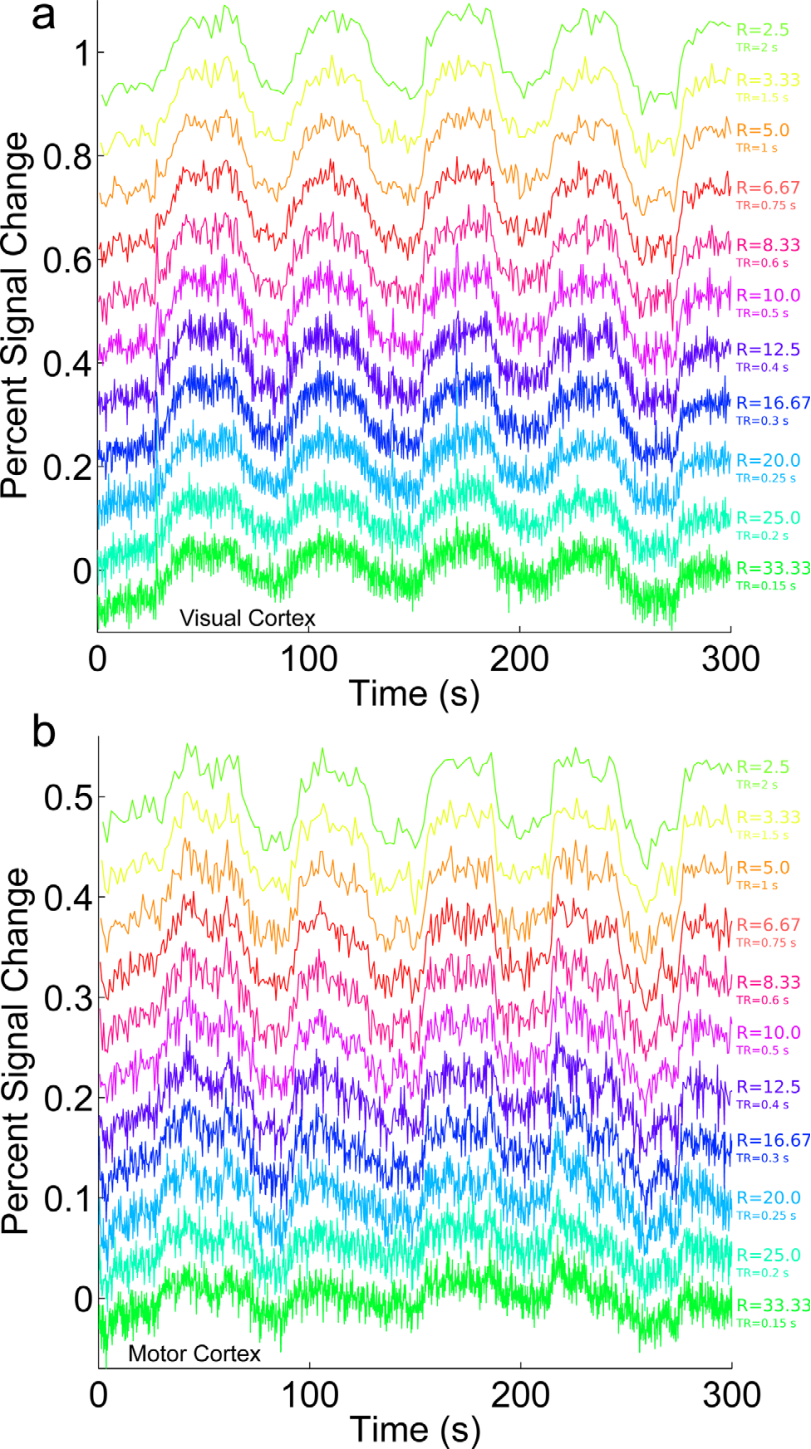
Example voxel time‐series from representative high z‐stat voxels in the visual‐motor task reconstruction a voxel from visual cortex (**a**) and a voxel from motor cortex (**b**).

For the in vivo visual‐motor experiment, Figure 5 plots representative time‐series for voxels in visual and motor cortices from a single subject across multiple acceleration factors. A strong degree of similarity is exhibited across these reconstructions at vastly different temporal scales, which shows that the rank‐constrained reconstruction is capable of recovering dynamic temporal information at very high acceleration factor. Peristimulus plots showing block‐averaged hemodynamic responses at various acceleration factors can be found in Supporting Figure S2.

While no ground truth is available, slight loss of contrast occurs at higher acceleration factors (reduced percent signal changes), in both the visual and motor cortex data, although peak z‐statistics do not change notably (Sup. Fig. S3). The similarity of peak z‐stats across acceleration factors after correcting for temporal autocorrelation has been reported previously, and reflects the choice of analysis, where single‐regression analyses do not benefit from the increase in time‐points as much as multiple‐regression analyses (like ICA) do 38.

**Figure 6:**
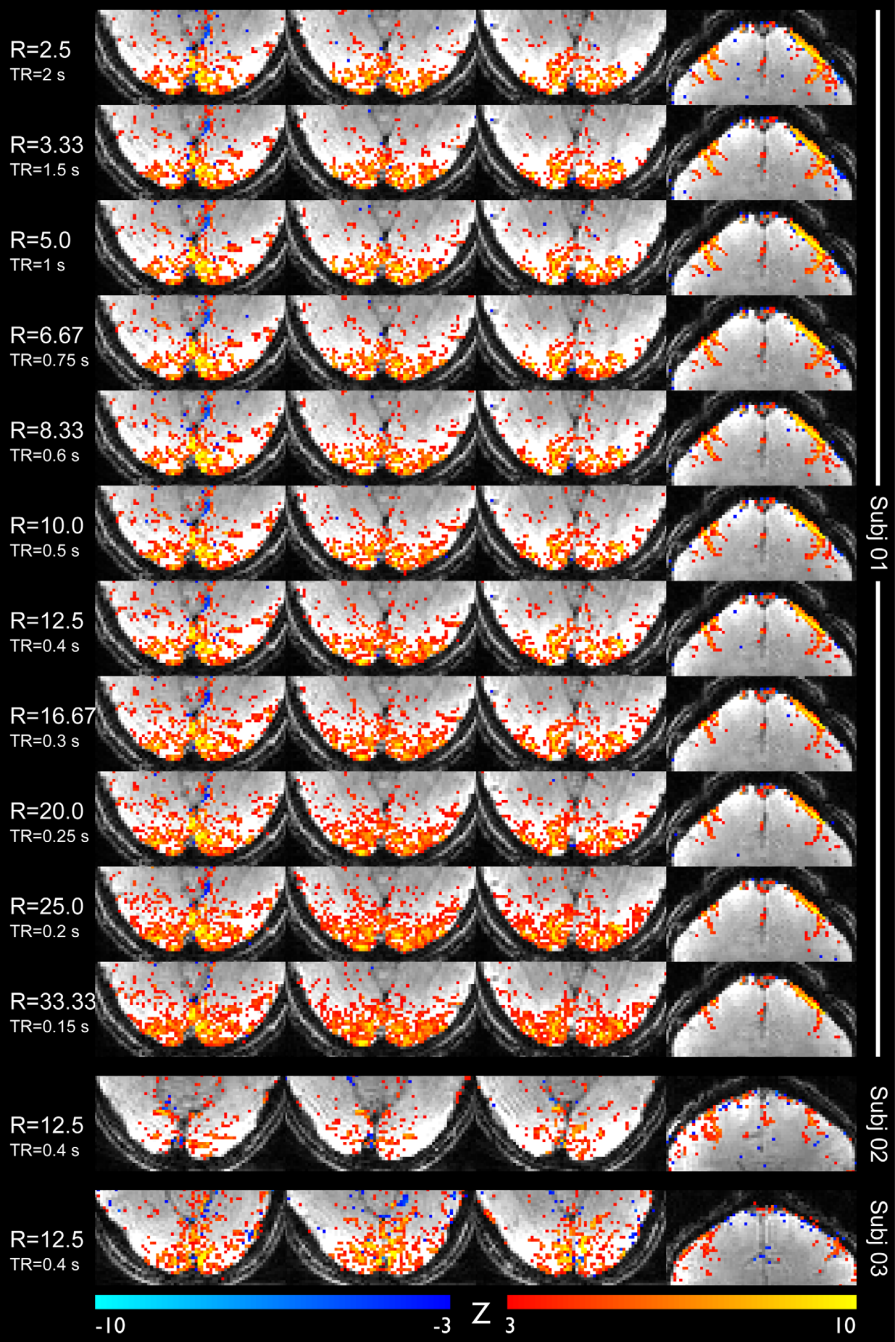
Activation maps highlighting activation in visual and motor cortex regions from subject 1 across acceleration factors, and from subjects 2 and 3 at R = 12.5. All maps were thresholded at |z|>3.

Figure 6 shows the results of the FEAT GLM analysis from all three subjects, in slices across the visual and motor cortices, overlaid on the T2* weighted images generated by k‐t FASTER. Similar to the time‐series results, z‐statistic spatial maps resulting from a regression analysis show a striking similarity across acceleration factors, indicating that the signal component corresponding to the visual‐motor task has sufficiently high variance to be well characterized at high acceleration factors. An additional comparison of task activation results using a fully 3D reconstruction, instead of slice‐by‐slice, can be found in Supporting Figure S4.

Qualitative assessment of the time‐series and statistical maps suggests that motor and visual activation is well characterized up to maximum acceleration factors around R = 12.5 without much loss of task contrast or activation. A quantitative analysis of the spatial fidelity of the statistical maps at different acceleration factors using a receiver‐operator characteristic (ROC) (taking the data at the R = 2.5 as representative of ground truth) found that total area under the ROC curve was as high as 0.99 at R = 12.5 and showed the steepest drop‐off after R = 16.67, consistent with the qualitative assessment (Sup. Fig. S5).

**Figure 7:**
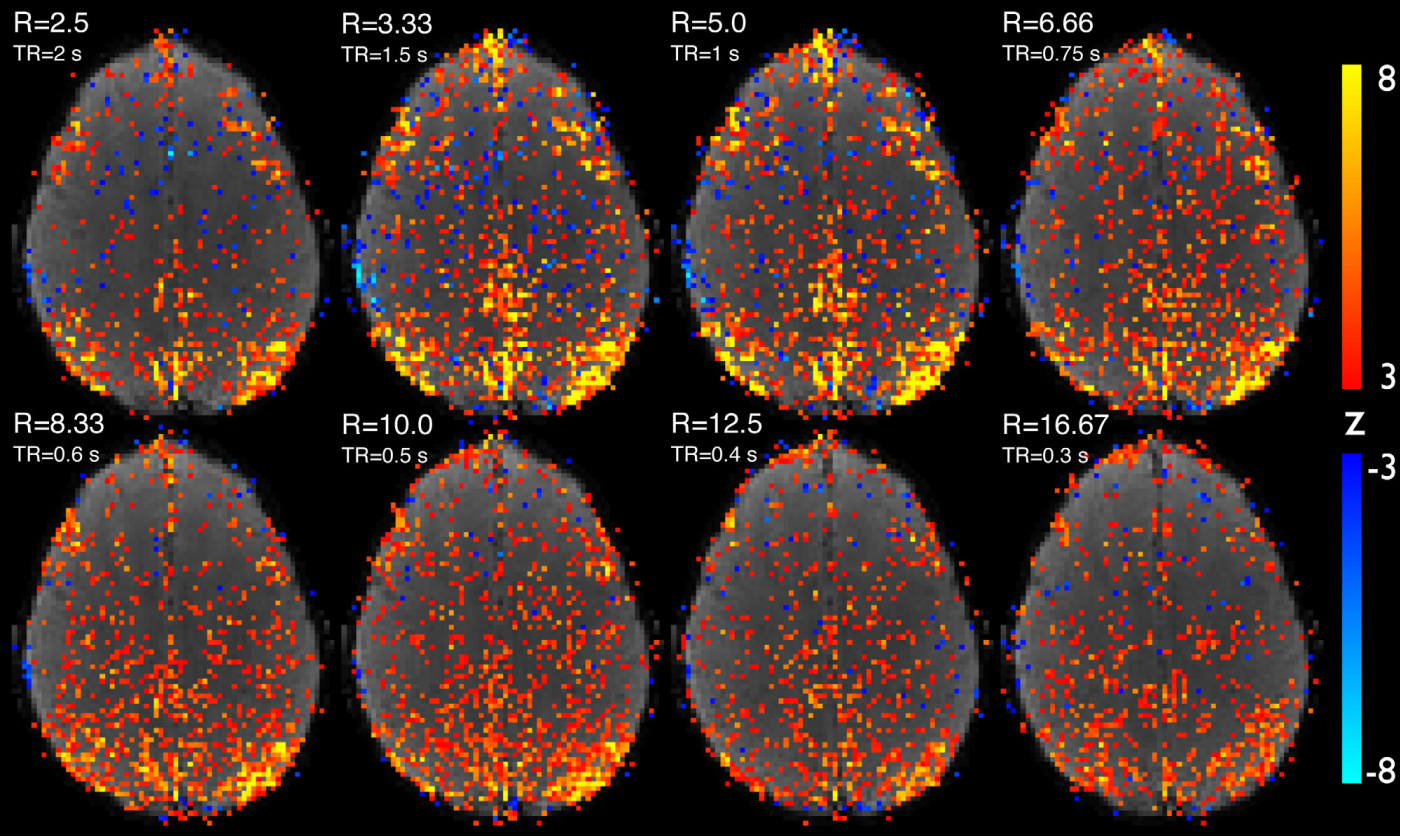
Default mode network expression from a single subject ICA in a visual‐motor dataset, at acceleration factors of R = 2.5 to R = 16.67. All maps are thresholded at |z| > 3.

In addition to looking at task components, we investigated the presence of intrinsic brain networks in subject 1 of the visual‐motor task experiment. Figure 7 shows the manually identified ICA spatial component corresponding to the task anticorrelated default mode network (DMN) 39, at acceleration factors of R = 2.5 to 16.67 (no DMN components were found in datasets at R > 16.67). The characteristic posterior parietal and anterior regions can be seen most clearly at R = 3.33 or 5. These results are consistent with the default mode network accounting for less variance than the task networks (Sup. Figs. S6, S7), and, therefore, showing lower peak tolerable acceleration factors, while also demonstrating the utility of acceleration in analyses like ICA, where the additional time‐points provided by the R = 3.33 or 5 data result in better recovery of the DMN than the R = 2.5 data with fewer time‐points. The DMN expression is also significantly less robust than the task activation networks, which might be expected due to the simple sensorimotor task, rather than a higher level cognitive task 40.

**Figure 8:**
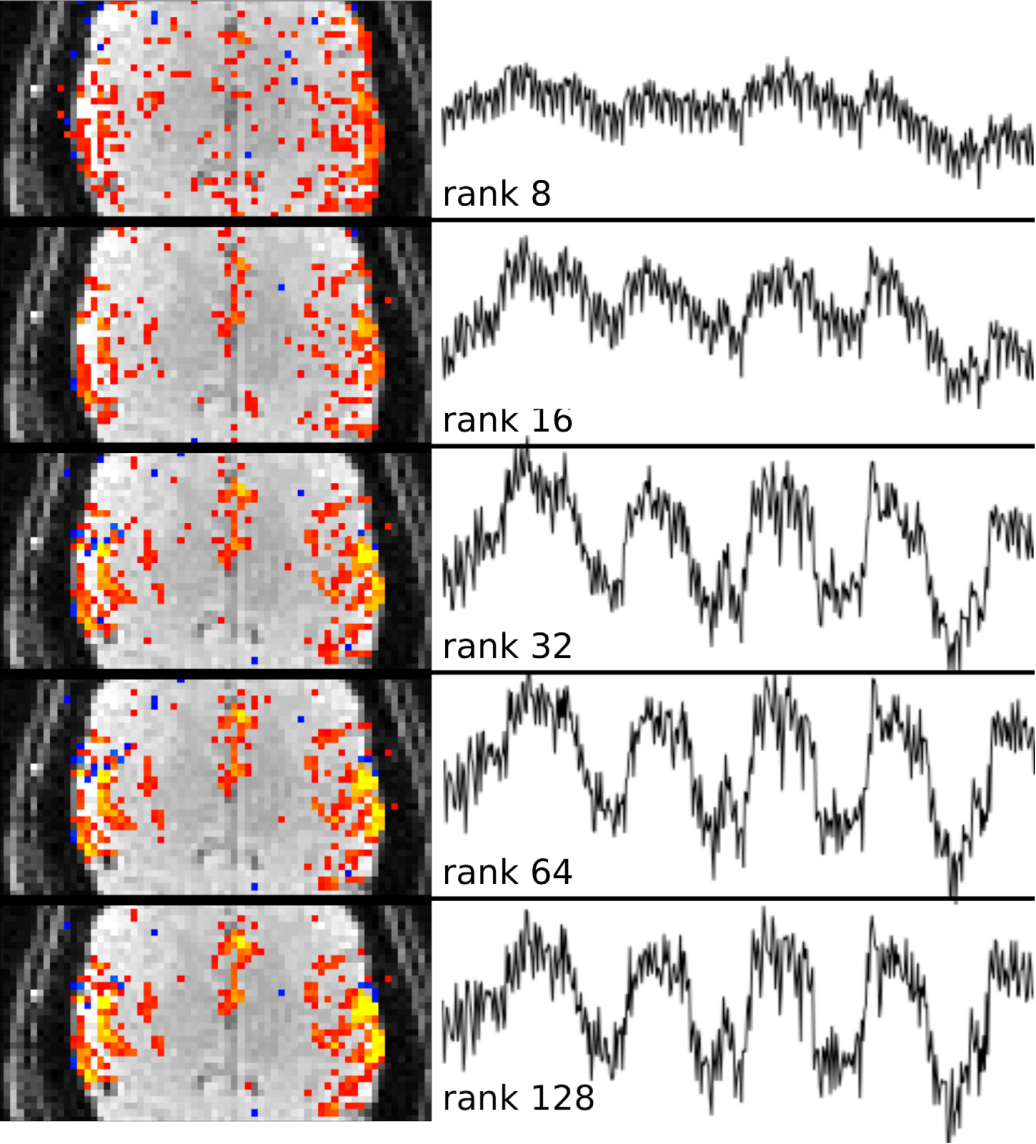
Activation maps and representative voxel time‐series for the visual‐motor experiment across different reconstruction rank constraints of 8, 16, 32, 64, and 128. Maps are thresholded at |z|>2.6.

The impact of the choice of rank constraint in the IHT+MS reconstruction algorithm can be seen in Figure 8, which shows activation and time‐series in motor cortex regions from subject 1 at R = 6.67 and reconstruction rank constraints of 8, 16, 32, 64, and 128. At rank 32 and beyond, the reconstructed data show little difference in spatial or temporal features. At ranks 8 and 16, however, it is apparent signal component corresponding to the visual‐motor task is not being captured well, suggesting that the signal components corresponding to the fMRI task are between 16 and 32 in variance order in the underlying data. At ranks of 64 and 128, there is little apparent penalty for selecting a rank constraint that overestimates the maximum dimensionality permitted by the degree of undersampling. Furthermore, the effective impact of the nonlinear reconstruction process on spatial fidelity is marginal, approximated by the size of an equivalent smoothing kernel that can be estimated by analyzing the spatial smoothness (autocorrelation) of the residuals after GLM fitting using Gaussian random field theory (Sup. Fig. S8).

**Figure 9:**
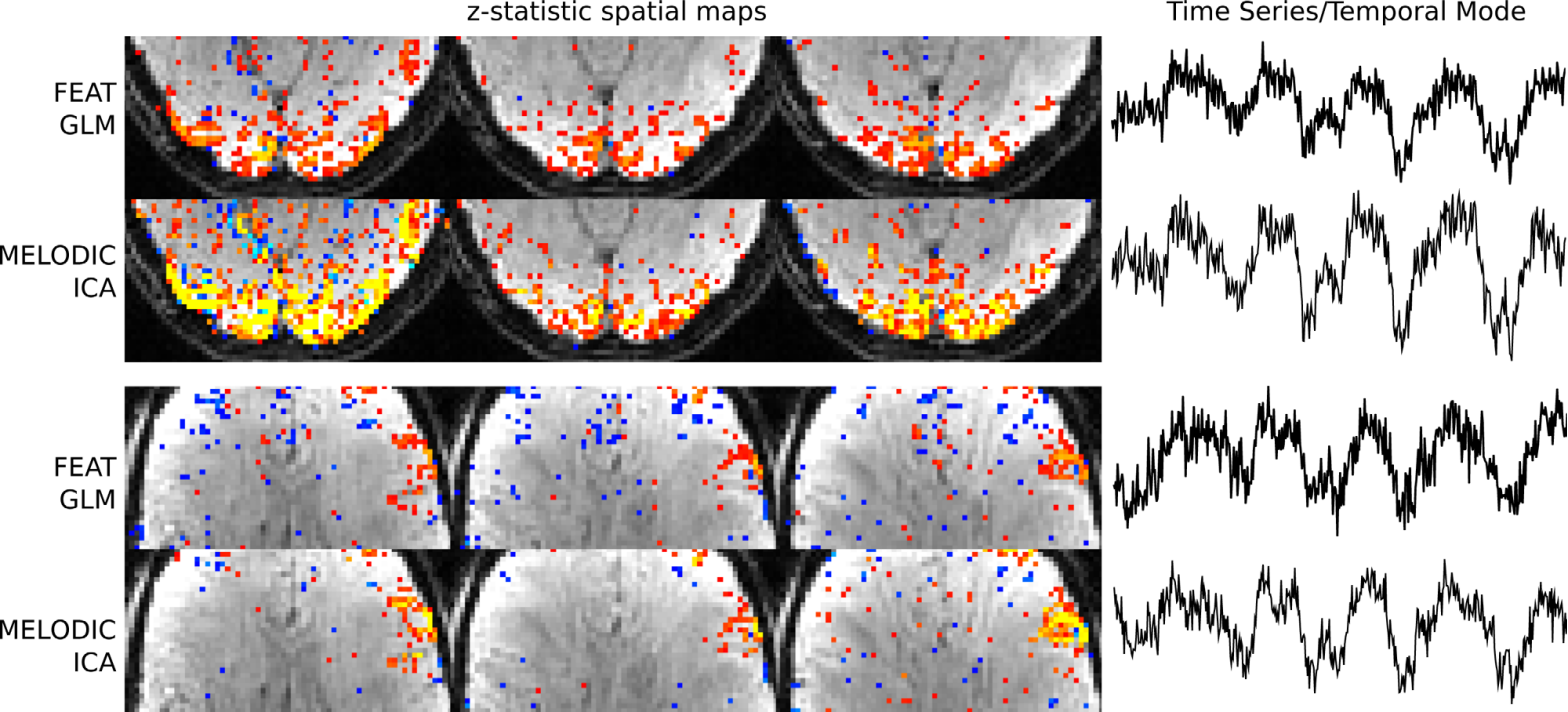
Activation maps from FEAT GLM and representative high z‐stat voxel time‐series (rows 1,3) and corresponding spatial component maps and temporal modes from a MELODIC ICA (rows 2,4), from one subject in the category fluency task. Rows 1 and 2 show activation in visual cortex, while rows 3 and 4 show activation in Broca's area. GLM maps are thresholded at |z|>2.6, and ICA maps are thresholded at |z|>4.

Finally, Figure 9 shows both GLM and ICA‐derived activation maps and time‐courses from one subject in the category fluency task at R = 8.33. The chosen acceleration factor reflects the qualitative limit on acceleration in this lower variance cognitive task. Here activation in visual cortex and Broca's area is evident, and peak z‐statistics for both subjects were 9.82 and 9.77 in the GLM analysis and 14.86 and 11.04 for the ICA component containing Broca's area. The ICA results show similar spatial patterns of activation, with temporal modes corresponding very well to the block design task.

## DISCUSSION

Using k‐t FASTER, we show that significant further improvements in data matrix reconstruction fidelity can be made using a hybrid radial‐Cartesian sampling scheme, and incorporating coil sensitivity encoding in the measurement operator. Task fMRI experiments showed that with these modifications, high acceleration factors of up to R = 12.5 or 16.67 in sensorimotor task fMRI can be achieved, whereas the category fluency task and intrinsic DMN recovery were found to exhibit lower maximum tolerable acceleration factors of R = 8.33 and R = 5, respectively.

The compressibility of fMRI data depends largely on context. In simple task‐based fMRI, the signals of interest are typically high variance, and therefore well described with a model with very low rank (and hence highly compressible). In contrast, intrinsic resting state network fluctuations can be subtle, with variances close to the noise floor, and need relatively higher rank models (permitting lower acceleration). These precise limits are subject‐specific, and also depend on the total sampling duration. As such, the utility of k‐t FASTER in recovery of subtle, low‐variance signals may not lie necessarily in reducing overall scan times, but rather to increase sampling rates for increased spatial resolution or temporal degrees of freedom. With high variance signals, however, acceleration factors approaching those obtained by InI methods 41 or MREG 42 is achievable. While sampling procedures are similar, k‐t FASTER does not exhibit the spatial fidelity loss characteristic of these methods that rely heavily on spatial regularization, instead of leveraging temporal domain information and spatiotemporal redundancy.

The EPI‐based sampling allows inverse Fourier transform along the EPI phase‐encoding direction, enabling efficient, parallelizable slice‐by‐slice reconstruction. Furthermore, the EPI readout provides additional utility in estimating global phase shifts associated with scanner drift and physiological fluctuations, and even physiologically induced linear phase shifts along the z‐direction on a shot‐by‐shot basis 22. Moreover, the flexible temporal resolution in the trajectory should enable complete estimation of rigid‐body motion from images reconstructed at very high temporal resolution from very few projections. Future work will consider the inclusion of such corrections to improve robustness to motion, as well as the estimation and removal of other artifacts such as physiological fluctuations before reconstruction of functional information.

The spatiotemporal flexibility of reconstruction may also be beneficial in tuning single‐subject experiments, as in presurgical planning. In this case, an acquisition scheme that does not impose a rigid relationship between spatial and temporal resolution may provide a greater opportunity to find the optimal balance between spatial fidelity and statistical power, without the constraint of fixing imaging parameters across a group or cohort. It also permits potentially exciting possibilities for performing separable reconstructions of the spatial and temporal information contained in fMRI data. For example, consider independent reconstructions, one optimized for high spatial fidelity, and another optimized for temporal fidelity. While some care must be taken to account for the possibility of different features being present at different scales, these data might be corelated or combined in several ways (e.g., by means of regression) to produce high fidelity spatiotemporal information that would otherwise not be accessible without this type of acquisition scheme.

One challenge of using a non‐Cartesian sampling scheme is in ensuring that the sampled trajectory is consistent with the expected, designed trajectory, particularly when high undersampling factors are used. In this work, trajectory shifts along the readout direction were corrected using back‐and‐forth running EPI navigator lines, as well as the abovementioned physiological phase corrections, but no additional trajectory calibration or corrections were used. Future work with hybrid radial‐Cartesian sampling or other optimized 3D trajectories may benefit from more sophisticated trajectory correction or mapping to ensure that data consistency constraints are enforced on self‐consistent k‐space data, to prevent over‐fitting of inconsistent measurement data.

The primary differences between the k‐t FASTER fixed‐rank reconstruction approach and other proposed rank‐based methods for fMRI acceleration are that (i) only rank constraints are used, and (ii) no prior information is required (aside from prior knowledge that the data matrix has a relatively low rank representation). In the method proposed by Lam et al 18, rank‐constrained reconstruction follows the partial separability imaging model 43, where the temporal subspace is first defined using signals from only the densely sampled portion of central k‐space. The spatial subspace is then estimated with an additional direct sparsity constraint on the spatial component vectors. Similarly, the fLORA method proposed by Nguyen and Glover 19 also uses the partial separability model, and uses additional group sparsity constraints in their reconstruction, as well as reference images. However, estimating the temporal subspace before image reconstruction from only a subset of the spatial domain may not be optimal due to the assumption that the central k‐space temporal subspace is valid for the full k‐space dataset 44; in our method, both the spatial and temporal subspaces are estimated simultaneously, from the entire set of samples. While regularization can improve the conditioning of inverse problems, sparsity constraints impose a specific model of data structure. Our approach aims to avoid potential estimation bias by not enforcing any structural constraints (spatial or temporal) on the data whatsoever.

The ICA results further demonstrate that recovery of task‐related and intrinsic network components is achieved using our relatively model‐free approach to reconstruction, and not simply driven by selective thresholding of simple regression analyses. Although the task designs used in the experiments were relatively simple and periodic, this information is not used or imposed in any way during the reconstruction, and we can expect the improved k‐t FASTER to perform equally well given arbitrary time courses, as we demonstrated in the simulations based on retrospectively sampled resting state data and in our previous work in estimation of resting state networks 11.

## CONCLUSIONS

With hybrid radial‐Cartesian sampling, and coil sensitivity encoding, k‐t FASTER is capable of highly accelerating fMRI data acquisition, enabling posthoc selection of acceleration factors dependent on the desired output model order, while maintaining high spatial and temporal fidelity. The generality of the k‐t FASTER approach makes it suitable for both task‐based and resting‐state fMRI, with no differences in reconstruction aside from selection of algorithm parameters.

## Supporting information


**Fig. S1.** Comparison of representative images from ground truth (**a**), k‐t FASTER (**b**), and conjugate gradient SENSE (**c**) reconstructions. While the k‐t FASTER image is nearly indistinguishable from ground truth, noticeable blurring and residual streaking artifacts (particularly near the posterior end of the image) is evident, highlighting the benefit of the rank constraint in addition to enforcing multicoil consistency. Similarly, (**d**) shows a time‐series from a representative voxel, highlighting the excellent correspondence of the k‐t FASTER time‐course (red) with ground truth (blue), whereas the conjugate gradient SENSE time‐course (yellow) is quite noisy. In (**e**), a 10 × zoomed portion of (d) is shown, and in both (d,e) plots are offset vertically for display clarity. Note that these time‐series do not reflect task activation, but a resting‐state time‐course from the center of the brain.
**Fig. S2.** Peristimulus plots from subject 1 in the visual‐motor experiment, across all tested acceleration factors. Masks were drawn manually over visual and motor cortices, and data for all plots were averaged over an additional z‐statistic mask defined by the intersection across all acceleration factors of all voxels with z‐stats > 2.3.
**Fig. S3.** Peak z‐statistics from the visual‐motor task across acceleration factors of R = 2.5 to R = 16.67, from all three subjects. Regions of interest around the primary visual and the primary motor areas were drawn for each subject, with peak z‐stats reported separately for each region. Although some variation is present, the general trend is roughly consistent with consistent peak z‐stats at varying acceleration factors for GLM analyses. Deviations from this may be due to poor fitting of mixture models that produce the corrected z‐statistic distributions.
**Fig. S4.** ICA spatial maps, time‐series and power spectra for a representative 3D reconstruction (in subject 1) at R = 5. The left column represents the visual component, containing 2.10% of the total variance in the data, and the right column represents the motor component, containing 1.01% of the total variance in the data. These maps can be directly compared with the visual and motor components in Supporting Figure S6. In this 3D reconstruction, the entire 3D+time matrix was reconstructed at the same rank constraint of the slice‐by‐slice 2D+time matrices, 32, which was sufficient to capture the task components, but not sufficient to recover the intrinsic default mode network well.
**Fig. S5.** Top Row: Receiver operator characteristics (ROC) for subject 1 in the visual‐motor task data, across the entire volume (first column), visual areas (second column) and motor regions (third column). Bottom Row: Area under the ROC curves. The ROC results are generated based on using the R = 2.5 (thresholded at |z|>3) data as ground truth, as no actual ground truth is available. In the overall dataset, the area under the ROC curve is 0.99 at R = 12.5.
**Fig. S6.** ICA spatial maps, time‐series and power spectra for subject 1 at R = 5. The left column represents the visual component, containing 2.76% of the total variance in the data, and the right column represents the motor component, containing 1.17% of the total variance in the data. These maps can be directly compared with the visual and motor components derived from the 3D reconstruction in Fig. S5, as well as the GLM results in Figures 4 and 5. While here the motor component appears to have significantly lower variance, it is apparent that the mainly visual component does have motor representation as well, so this left column component may be better characterized as a visual‐motor component, and the right column as a secondary motor component.
**Fig. S7.** ICA spatial map, time‐series and power spectrum for subject 1 at R = 5. This data correspond to the default mode network, containing 0.87% of the total variance in the data. One feature that is evident is that most of the high z‐stats in this component are confined in the z‐direction to the superior portion of the brain, which is likely a consequence of the 2D separable slice‐independent reconstruction, in which the slice‐by‐slice enforcement of rank constraints acts like a z‐dependent filtering or dimensionality reduction.
**Fig. S8.** This shows that spatial resolution decreases monotonically with acceleration, and acceleration factors of R = 5–8.33 have effective kernel FWHM sizes that are approximately equal to the nominal voxel dimension of 2 mm. However, this effect of resolution loss is modest, showing only ∼10–15% resolution loss at R = 12.5, up to ∼30% increase in effective voxel size at R = 33.33.
