# Supplementary material for: Accelerating functional MRI using fixed‐rank approximations and radial‐cartesian sampling
Source: Magn Reson Med. 2016 Jan 17;76(6):1825–36. doi: 10.1002/mrm.26079 (PMC4847647; doi:10.1002/mrm.26079)
Supplement: Supplementary file 1 — Fig. S1. Comparison of representative images from ground truth (a), k‐t FASTER (b), and conjugate gradient SENSE (c) reconstructions. While the k‐t FASTER image is nearly indistinguishable from ground truth, noticeable blurring and residual streaking artifacts (particularly near the posterior end of the image) is evident, highlighting the benefit of the rank constraint in addition to enforcing multicoil consistency. Similarly, (d) shows a time‐series from a representative voxel, highlighting the excellent correspondence of the k‐t FASTER time‐course (red) with ground truth (blue), whereas the conjugate gradient SENSE time‐course (yellow) is quite noisy. In (e), a 10 × zoomed portion of (d) is shown, and in both (d,e) plots are offset vertically for display clarity. Note that these time‐series do not reflect task activation, but a resting‐state time‐course from the center of the brain. Fig. S2. Peristimulus plots from subject 1 in the visual‐motor experiment, across all tested acceleration factors. Masks were drawn manually over visual and motor cortices, and data for all plots were averaged over an additional z‐statistic mask defined by the intersection across all acceleration factors of all voxels with z‐stats > 2.3. Fig. S3. Peak z‐statistics from the visual‐motor task across acceleration factors of R = 2.5 to R = 16.67, from all three subjects. Regions of interest around the primary visual and the primary motor areas were drawn for each subject, with peak z‐stats reported separately for each region. Although some variation is present, the general trend is roughly consistent with consistent peak z‐stats at varying acceleration factors for GLM analyses. Deviations from this may be due to poor fitting of mixture models that produce the corrected z‐statistic distributions. Fig. S4. ICA spatial maps, time‐series and power spectra for a representative 3D reconstruction (in subject 1) at R = 5. The left column represents the visual component, containing 2.10% of the tot [file MRM-76-1825-s001.pdf]

Figure S1

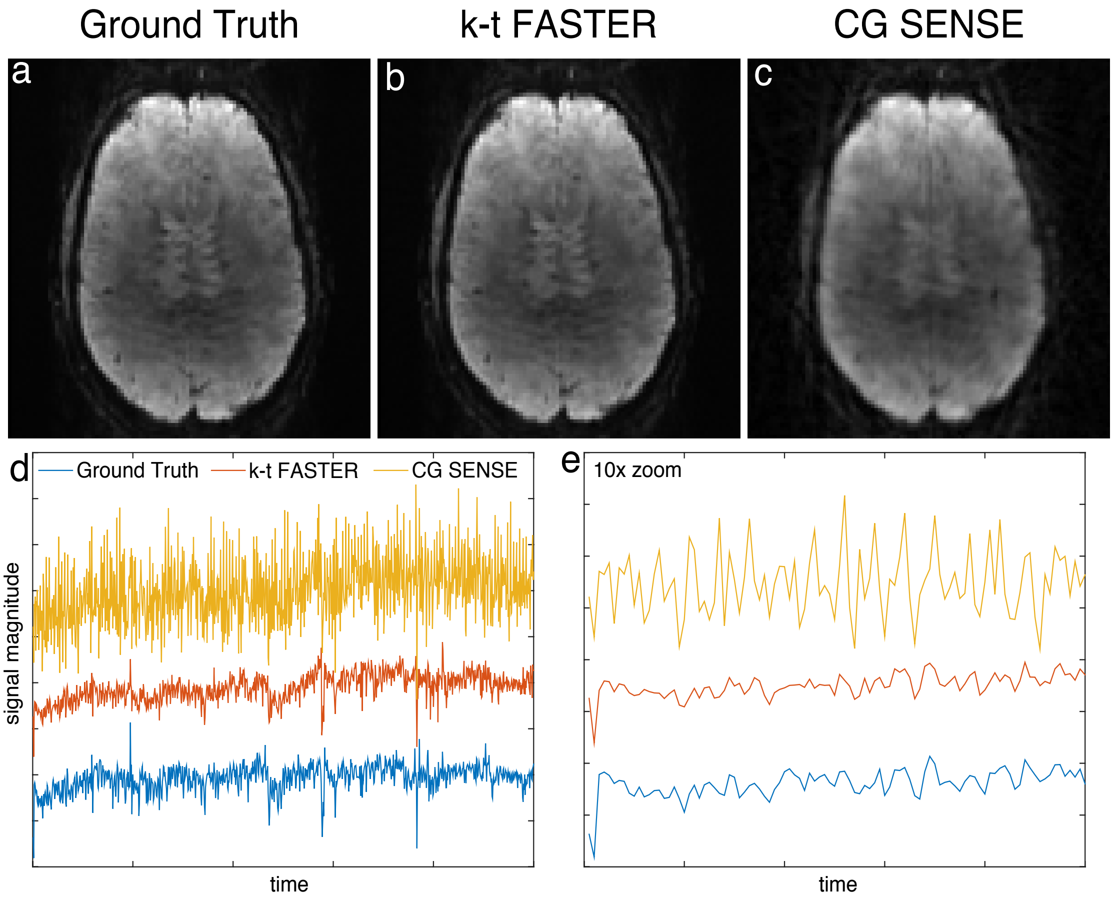

Figure S1 – Comparison of representative images from (a) ground truth, (b) k-t FASTER, and (c) conjugate gradient SENSE reconstructions. While the k-t FASTER image is nearly indistinguishable from ground truth, noticeable blurring and residual streaking artefacts (particularly near the posterior end of the image) is evident, highlighting the benefit of the rank constraint in addition to enforcing multi-coil consistency. Similarly, (d) shows a time-series from a representative voxel, highlighting the excellent correspondence of the k-t FASTER time-course (red) with ground truth (blue), whereas the conjugate gradient SENSE time-course (yellow) is quite noisy. In (e), a 10x zoomed portion of (d) is shown, and in both (d,e) plots are offset vertically for display clarity. Note that these time-series do not reflect task activation, but a resting-state time-course from the centre of the brain.

Figure S2

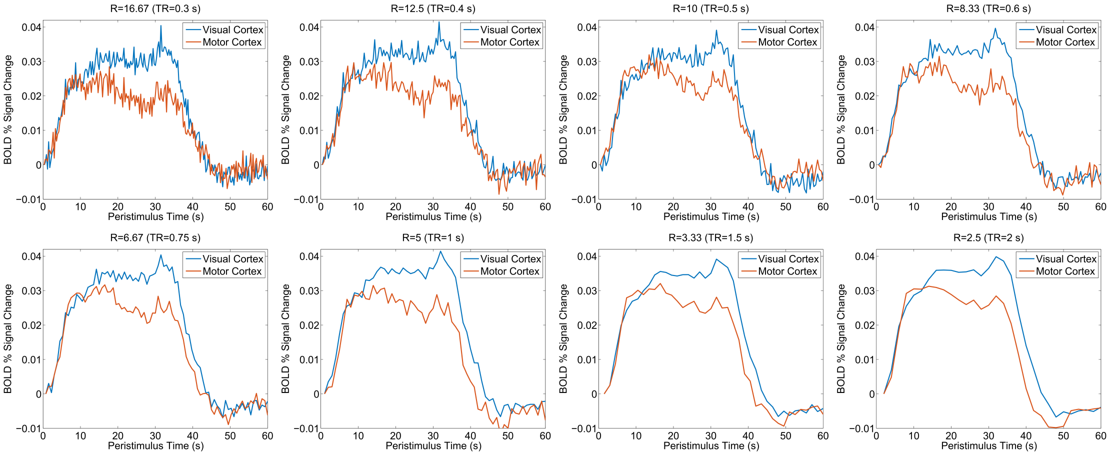

Figure S2 – Peri-stimulus plots from subject 1 in the visual-motor experiment, across all tested acceleration factors. Masks were drawn manually over visual and motor cortices, and data for all plots were averaged over an additional z-statistic mask defined by the intersection across all acceleration factors of all voxels with z-stats > 2.3.

Figure S3

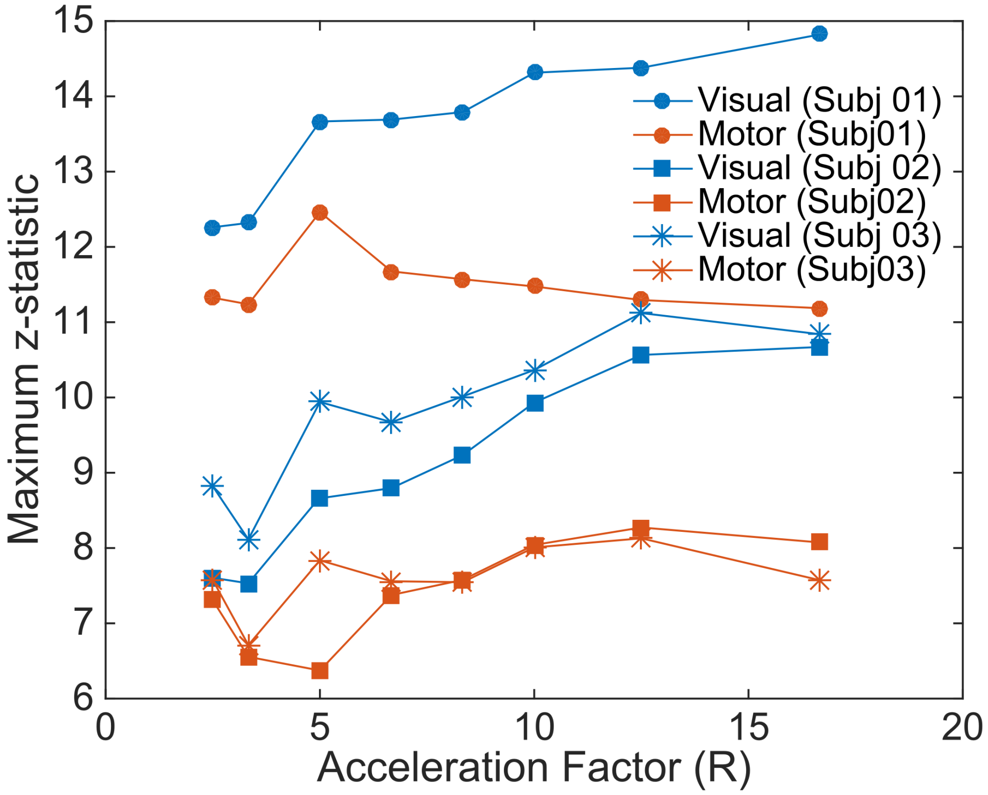

Fig. S3 – Peak z-statistics from the visual-motor task across acceleration factors of R=2.5 to R=16.67, from all three subjects. Regions of interest around the primary visual and the primary motor areas were drawn for each subject, with peak z-stats reported separately for each region. Although some variation is present, the general trend is roughly consistent with consistent peak z-stats at varying acceleration factors for GLM analyses. Deviations from this may be due to poor fitting of mixture models that produce the corrected z-statistic distributions.

Figure S4

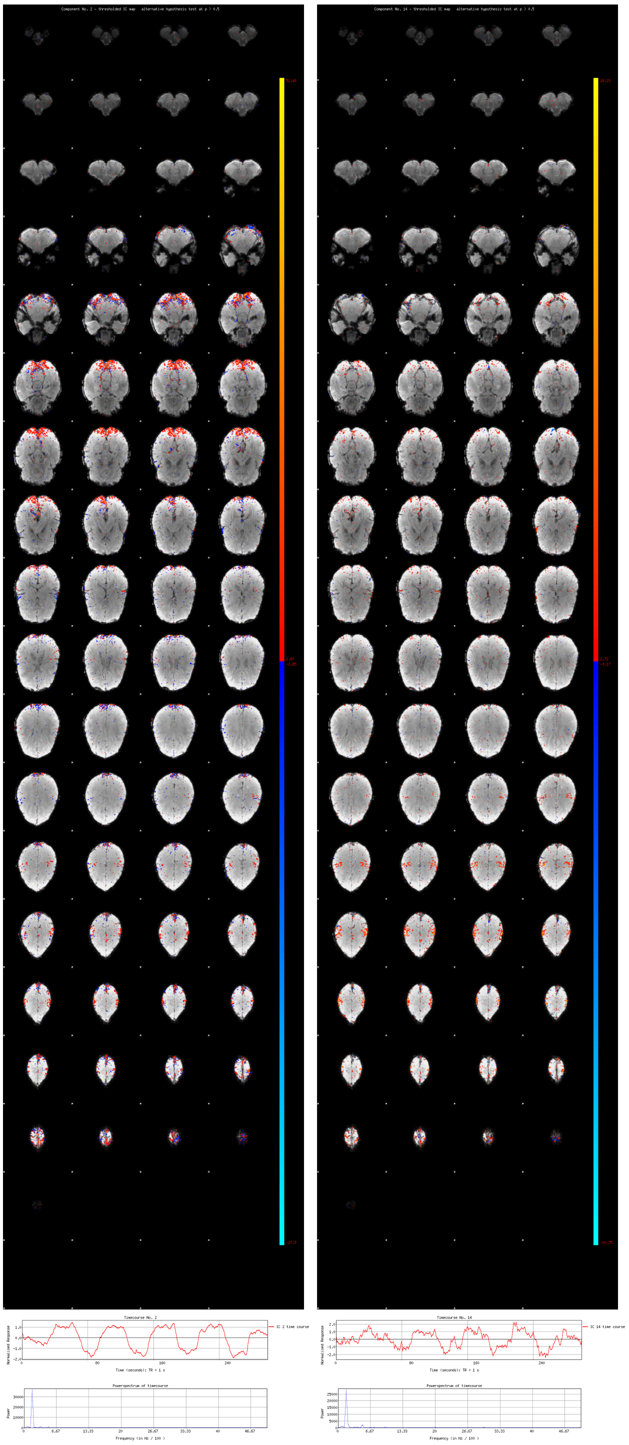

Figure S4 – ICA spatial maps, time-series and power spectra for a representative 3D reconstruction (in subject 1) at R=5. The left column represents the visual component, containing 2.10% of the total variance in the data, and the right column represents the motor component, containing 1.01% of the total variance in the data. These maps can be directly compared to the visual and motor components in Fig. S6. In this 3D reconstruction, the entire 3D+time matrix was reconstructed at the same rank constraint of the slice-by-slice 2D+time matrices, 32, which was sufficient to capture the task components, but not sufficient to recover the intrinsic default mode network well.

Figure S5

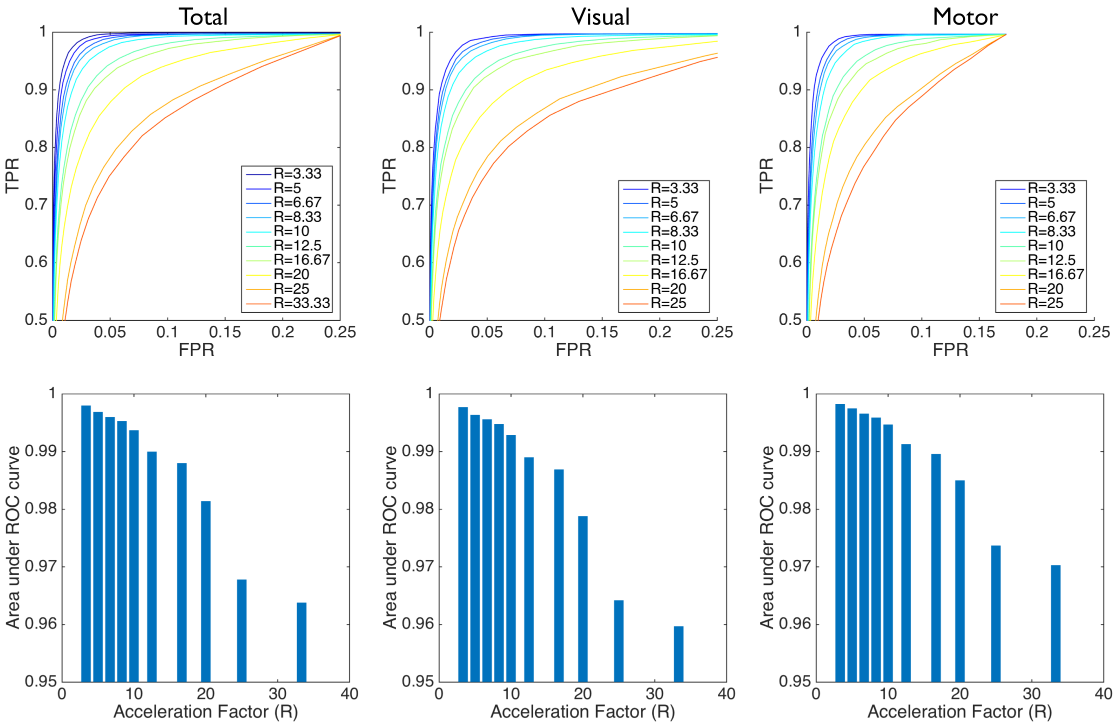

Fig S5 – Top Row: Receiver operator characteristics (ROC) for subject 1 in the visual-motor task data, across the entire volume (first column), visual areas (second column) and motor regions (third column). Bottom Row: Area under the ROC curves. The ROC results are generated based on using the R=2.5 (thresholded at |z|>3) data as ground truth, as no actual ground truth is available. In the overall dataset, the area under the ROC curve is 0.99 at R=12.5.

Figure S6

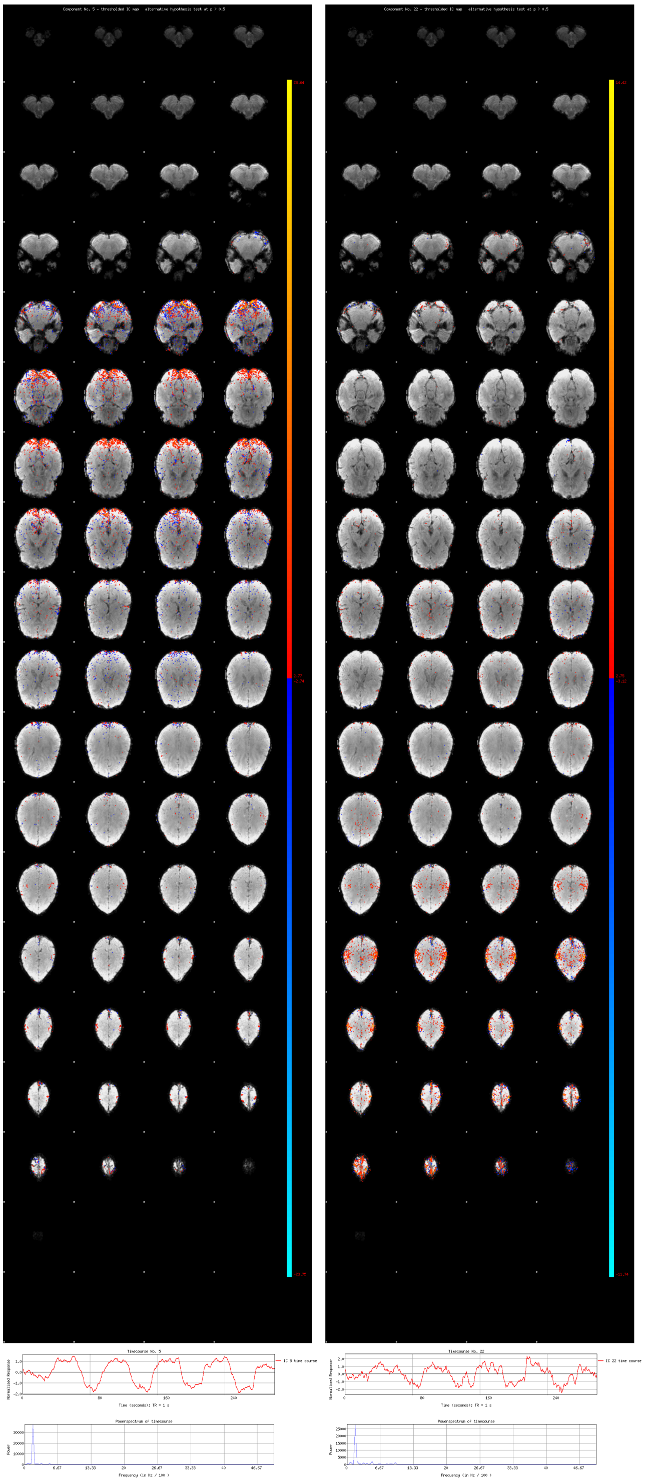

Figure S6 – ICA spatial maps, time-series and power spectra for subject 1 at R=5. The left column represents the visual component, containing 2.76% of the total variance in the data, and the right column represents the motor component, containing 1.17% of the total variance in the data. These maps can be directly compared to the visual and motor components derived from the 3D reconstruction in Fig. S5, as well as the GLM results in Figs. 4 and 5. While here the motor component appears to have significantly lower variance, it is apparent that the mainly visual component does have motor representation as well, so this left column component may be better characterised as a visual-motor component, and the right column as a secondary motor component.

<u>**Figure S7**</u>

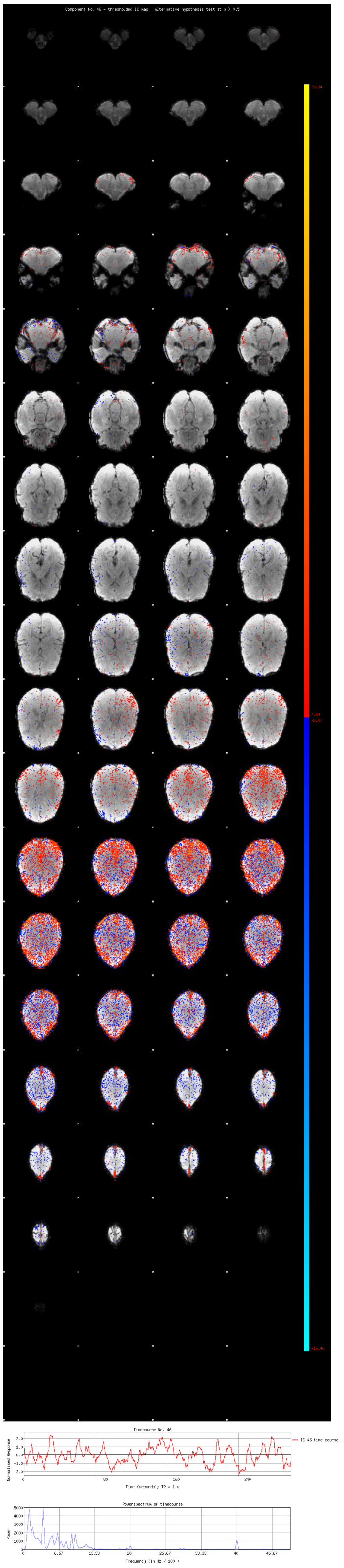

Figure S7 – ICA spatial map, time-series and power spectrum for subject 1 at R=5. This data correspond to the default mode network, containing 0.87 % of the total variance in the data. One feature that is evident is that most of the high z-stats in this component are confined in the z-direction to the superior portion of the brain, which is likely a consequence of the 2D separable slice-independent reconstruction, in which the slice-by-slice enforcement of rank constraints acts like a z-dependent filtering or dimensionality reduction.

Figure S8

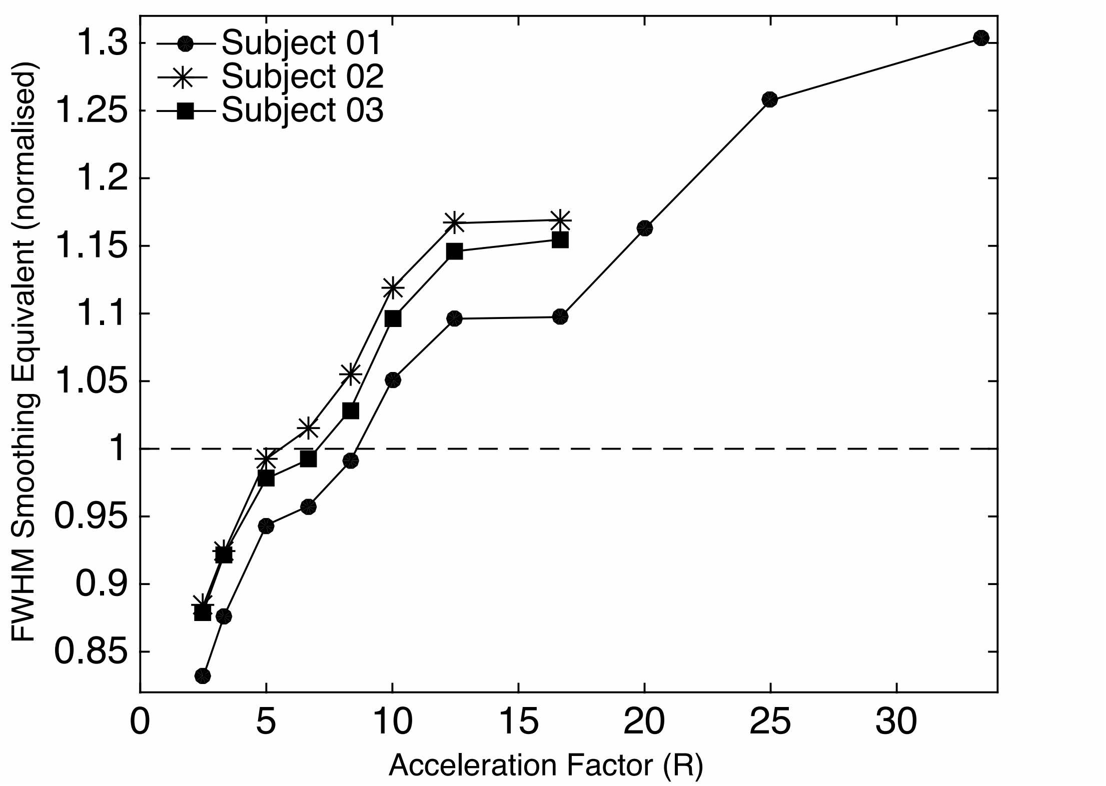

Figure S8 – This shows that spatial resolution decreases monotonically with acceleration, and acceleration factors of R=5 – 8.33 have effective kernel FWHM sizes that are approximately equal to the nominal voxel dimension of 2 mm. However, this effect of resolution loss is modest, showing only ~10-15% resolution loss at R=12.5, up to ~30% increase in effective voxel size at R=33.33.
